# IUC24412-78 The safety and efficacy of a modified Di stasi regime for patients with non-muscle invasive bladder cancer during times of bacillus Calmette-Guérin shortage

**DOI:** 10.1093/oncolo/oyaf248.018

**Published:** 2025-08-29

**Authors:** P Carter, L Nicholson, J Borwell, J Brewin

**Affiliations:** Salisbury NHS Foundation Trust, United Kingdom; Salisbury NHS Foundation Trust, United Kingdom; Salisbury NHS Foundation Trust, United Kingdom; Salisbury NHS Foundation Trust, United Kingdom

## Abstract

**Background:**

Intravesical Bacillus Calmette-Guérin (BCG) is an important treatment for non-muscle invasive bladder cancer (NMIBC). BCG combined with electromotive drug administration (EMDA) mitomycin C (MMC) can decrease the risk of bladder cancer recurrence and disease progression when compared with BCG monotherapy. The standard Di Stasi induction protocol involves 9 weeks of alternating intravesical BCG and EMDA MMC instillations. Due to global BCG shortages, exacerbated by the COVID-19 pandemic, modified regimes have been implemented. We sought to evaluate the safety and efficacy of a modified Di Stasi regimen during a period of BCG shortage.

**Methods:**

We conducted a retrospective cohort analysis of 40 consecutive patients being treated for high-risk NMIBC between 2021 and 2022. Nineteen patients completed a modified Di Stasi regime (3 BCG and 6 EMDA MMC) compared to 21 patients who completed the standard regime (6 BCG and 3 EMDA MMC). Patient demographics, histology and recurrence rate over 3 years were collected from the electronic patient records and analyzed.

**Results:**

A chi-square test showed no significant difference in characteristic data between the 2 cohorts. Recurrence-free survival at 36 months was analyzed using Kaplan–Meier survival analysis and was 71.4% in the standard Di Stasi cohort compared to 62.2% in the modified Di Stasi cohort. The Log-rank method yielded a non-significant difference in the rate of recurrence-free survival at 36 months (*P* = .638). The mean time to recurrence was significantly longer in the modified Di Stasi cohort, 29.7 ± 2.6 months vs 20.9 ± 1.6 months (*P* < .0001). Total recurrence in the modified Di Stasi group was 36.8% (*n* = 7) and 30.0% (*n* = 6) in the standard Di Stasi cohort (*P* = .59). Mortality from bladder cancer at 36 months between the 2 groups was not significant, 10.5% (*n* = 2) in the modified Di Stasi vs 0.0% (*n* = 0) in the standard Di Stasi (*P* = .13).

**Conclusions:**

This modified Di Stasi protocol demonstrated comparable recurrence-free survival at 36 months and a prolonged mean time to recurrence. In future periods of BCG shortage, a modified regime may be considered as an alternative to the conventional Di Stasi protocol and offer similar clinical outcomes.

